# The caregiving relationship and quality of life among partners of stroke survivors: A cross-sectional study

**DOI:** 10.1186/1477-7525-9-29

**Published:** 2011-05-09

**Authors:** Christine J McPherson, Keith G Wilson, Livia Chyurlia, Charles Leclerc

**Affiliations:** 1 School of Nursing, Faculty of Health Sciences, University of Ottawa, 451, Smyth Road, Ottawa, Ontario, K1H 8M5, Canada; 2 School of Psychology, Faculty of Social Sciences, University of Ottawa, 200 Lees Avenue, Ottawa, Ontario, K1N 6N5, Canada; 3 The Ottawa Hospital Rehabilitation Centre, 505 Smyth Road, Ottawa, Ontario, ON K1H 8M2, Canada; 4 Faculty of Medicine, University of Ottawa, 451, Smyth Road, Ottawa, Ontario, K1H 8M5, Canada

**Keywords:** Stroke, caregiving, quality of life, reciprocity, family, burden

## Abstract

**Background:**

Since the majority of stroke survivors return home following their stroke, families play a pivotal role in their care. Few studies have addressed both positive and negative aspects of this role or the broader construct of health-related quality of life (HRQL). Furthermore, little consideration has been given to the context of care in terms of relationship quality, and reciprocity. The present study examined the relationships between caregiver quality of life (HRQL), caregiver role, relationship satisfaction, balance and reciprocity in caregivers of partners who had experienced a stroke. Specific hypotheses were made based on equity theory in social relations.

**Methods:**

Fifty-six partner caregivers completed a postal survey that included measures of HRQL (SF-36), caregiver role (negative and positive aspects), relationship satisfaction, reciprocity and balance. Data were also collected on the care recipients' quality of life (Stroke Specific Quality of Life scale).

**Results:**

Compared to a normative sample, caregivers' HRQL was lower for all SF-36 domains. Care recipient and caregiver age, care recipient quality of life and caregiver role (negative) significantly predicted physical component summary scores on the SF-36, while care recipient quality of life and caregiver role (negative) significantly correlated with mental component summary scores. Relationship satisfaction and intrinsic rewards of caregiving were found to be important predictors of positive aspects of the caregiver role. Caregivers who viewed their relationship as less balanced in terms of give and take had significantly greater caregiver burden than those who viewed their relationship as more equitable.

**Conclusions:**

The study highlights the importance of taking a broader approach to examining partner caregiving in the context of stroke, in terms of the caregiving relationship and their influence on the health and well-being of caregivers.

## Background

Attending to the needs of a family member who has suffered a stroke, has been the subject of considerable research [1-5]. Similar to other areas of caregiving the emphasis has been on burden associated with this role, conceptualized predominantly in terms of emotional distress, social disruptions and economic limitations [1-3]. Fewer studies, however, have investigated the more global construct of quality of life in caregivers of a family member following stroke. Yet, an understanding of how caregiving influences elements such as life satisfaction, psychological, social, and physical functioning is important in fully appreciating the impact of caregiving [3,6].

Of those studies that have been conducted, in general, the findings indicate that caregiving negatively impacts on quality of life [1-3]. Based on White et al.'s (2004) conceptual model of quality of life for family caregivers of stroke survivors, the caregiving situation (e.g. care recipient dependency, impairment) is thought to directly and indirectly influence caregivers' quality of life. Indirectly, the demands of the caregiving situation are influenced by environmental factors (e.g. relationship, finances, support) and caregiver factors (e.g. personal attributes, perceived burden, health) [6]. For example, appraising caregiving as restrictive and burdensome can negatively affect caregiver quality of life [7], whereas, caregiver confidence in their knowledge about providing care and self-efficacy in the caregiving role have been found to be positively related to caregivers' quality of life in the domains of vitality (energy and fatigue) and mental health [8].

The latter finding relates to a smaller but growing body of literature examining the positive aspects of caregiving; an area that has been largely neglected in the caregiving literature [9]. Yet studies reveal that providing care to a family member can decrease anxiety and depression, increase a sense of fulfillment and self-esteem, and bring greater closeness in the relationship [10-12]. Indeed, qualitative studies where the caregiving role is explored in more depth have identified more of the positive aspects to this role [4,5]. Thus recognition that caregiving burdens and rewards can co-exist [13], adds to the importance of understanding both the positive and negative aspects of this experience in caregivers of stroke survivors, and how these roles relate to quality of life.

### Quality of the relationship

Cartwright, Archbold, Stewart and Limnadri (1994) emphasize the importance of a positive quality relationship in finding enrichment from caregiving [14]. The importance of the dyadic relationship cannot be overstated since caregiving occurs within the context of ongoing relationships between family members and adjustment to the impact of stroke brings about major changes and transformations in relationships [15,16]. In the psychological literature, relationship satisfaction has been shown to moderate the effects of anxiety as a consequence of caregiving [17]. Evidence from caregivers of older frail family members also shows that caregivers in relationships characterized as high in mutuality and closeness report lower levels of caregiver strain [18,19]. Moreover, active helping in relationships high in mutuality and interdependence can have positive effects for the care provider [20]. Surprisingly few quantitative studies however, have investigated the relationship on caregiver outcomes in people with stroke [21,22].

### Equity in the relationship

Relatedly, reflections of mutuality such as reciprocity are important in finding satisfaction and meaning in the caregiving relationship [14,23]. Equity theory in social relations is useful in understanding reciprocal exchanges between caregiver and care recipient [24,25]. Equity theory posits that individuals strive to maintain balance between benefits (receiving help and support) and contributions (giving help and support) within their relationships. Inequity arises when individuals give more than they receive (*underbenefit*) or receive more than they give (*overbenefit*). For example, when one member of the dyad becomes ill and is reliant on the other member for care then the balance may become inequitable. Equity theory predicts that inequity is psychologically uncomfortable for both members of the dyad; therefore, members are motivated to restore balance. Studies have identified that inequity in relationships can lead to negative emotions [26-28]

### Reciprocal exchanges

Within caregiving relationships there is the implicit assumption that exchanges of support are unidirectional flowing from caregiver to passive care recipient. However, even when a family member is ill and it becomes difficult to reciprocate with material tangible support, there is evidence that exchanges of a more interpersonal nature such as love, warmth and affection may still occur and be important for caregiver well-being [14,18,27,29]. Gleason and colleagues found that providing support within the context of a relationship characterized by exchanges of emotional support positively enhanced mood [27]. There is also evidence to suggest that reciprocity may decrease caregiver burden [18,28,29]. In a study of caregivers of frail older adults with illness or disability, Reid et al. found that exchanges of respect, regard and commitment between caregiver and care recipient, and balance in caregiving between caregiver and other family members were associated with lower levels of developmental, physical, social and emotional caregiver burdens [29]. To date however, little is known about the interactional aspects of the relationship in relation to caregiver outcomes such as caregiver role and health-related quality of life in caregivers of a family member following a stroke.

### Aims

To address the aforementioned gaps in the existing literature, the present study aims to test the following research hypotheses based on equity theory:

*Hypothesis 1 *- Caregiver perceived reciprocity will be significantly related to positive aspects of the caregiver role and inversely related to negative aspects of the caregiver role

*Hypothesis 2 *- Satisfaction with the relationship will be related to balance in give and take

*Hypothesis 3 *- Caregivers who are underbenefiting in the current relationship will have significantly higher scores on the caregiver role negative dimension compared to those who are equitable/overbenefiting from the relationship.

Secondary aims are: (1) to examine the relationship between HRQL, caregiver role, relationship satisfaction and reciprocity in family caregivers of partners with stroke, (2) to examine predictors of positive and negative aspects of the caregiver role and HRQL, and (3) compare caregivers HRQL to a normative non-caregiving sample.

## Methods

### Design

A cross-sectional survey design with a major correlational component was used. The research is part of a larger project examining family caregiving for individuals following stroke. Findings from the main study have been presented elsewhere [26].

### Research ethics

Research ethics approval was granted from the University of Ottawa Research Ethics Board and the Ottawa Hospital Rehabilitation Centre Research Ethics Board.

### Participants

Caregivers of former inpatients at the Ottawa Hospital Rehabilitation Centre were identified through medical charts. As part of the larger project, we identified consecutive patients between 2007-2008 with stroke whose hospital discharge scores on the Functional Independence Measure (FIM™) comprehension and communication items was ≥5; indicating a cognitive ability corresponding to the capacity to understand directions and communicate ideas with only standby prompting [30]. The inclusion criteria for caregivers were: that they were the partner of the care recipient; providing the majority of care at home; English or French speaking. To control for possible effects of the type of relationship on the outcomes under investigation the sample was limited to partner relationships.

From 120 eligible caregiver-care recipient dyads who were contacted to take part, 59 dyads responded (49.2% response rate). Incomplete care recipient (n = 2) and caregiver (n = 3) data meant that complete data was only available for 56 dyads. As shown in table 1, the majority of caregivers were female and were similar in age and educational level to care recipients. On average care recipients had experienced a stroke 31.7 months prior to the study.

**Table 1:** Participant Characteristics

		Caregiver (n = 56)*	Care recipient (n = 57)*
Age			
*Mean *(*SD*)		61.9 years (14.4)	65.2 years (14.6)
*Range*		29-88 years	32-88 years
Sex	Male	9 (16.1%)	50 (87.7%)
	Female	47 (83.9%)	7 (12.3%)
Education	More than high school	35 (62.5%)	34 (59.7%)
	High school	12 (21.4%)	11 (19.3%)
	Less than high school	8 (14.3%)	12 (21.1%)
Employment	Employed	19 (33.9%)	8 (14.0%)
	Retired/unemployed/medical leave	37 (66.1%)	49 (86.0%)
Help with care		18 (32.7%)	
Residing together		56 (100%)	
Time since stroke			
*Mean (SD) *			31.7 months (14.1)
*Range*			7-61 months
<6 months			0
6-12 months			18
>12 months			38
Stroke type	Infarct		40 (71.4%)
	Haemorrhage		14 (25.0%)
	Infarct and haemorrhage		2 (3.6%)
	Left hemisphere		29 (51.8%)
	Right hemisphere		24 (42.9%)
	Bilateral		3 (5.4%)

* The sum totals may vary due to missing data.

### Procedures and measures

A letter describing the study was sent from the medical director of the Ottawa Hospital Rehabilitation Centre stroke service to those eligible. Included with the letter was a pre-stamped postcard for caregivers to return if they chose not to participate. Two weeks later survey packages were sent to those who had not declined participation and again after four weeks if the surveys were not returned. After a further two weeks the research nurse contacted those who had not returned the survey to enquire if they were interested in taking part and whether they required assistance completing the survey. The research nurse conducted home visits to assist those requiring help to complete the surveys.

As part of the larger project care recipients provided demographic information and completed a measure of quality of life. These data were included in this study to allow comparisons between the variables and caregiver HRQL and role. Care recipients' quality of life was assessed using the Stroke-Specific Quality of Life Scale (SS-QOL) [31]. The SS-QOL is a reliable and valid disease-specific measure that assesses 12 domains pertinent to individuals with stroke (self-care, mobility, upper-extremity function, work/productivity, vision, language, thinking, personality, mood, energy, and family and social roles). The SS-QOL has three response formats, based on a 5-point scale: 1 = could not do it at all to 5 = no trouble at all, 1 = total help to 5 = no help needed, and 1 = strongly agree to 5 = strongly disagree. For the present study the overall score was used. This is derived from an unweighted average of the 12 domains. The internal consistencies for the 12 domains ranged from α = .77 to α = .95 in the current study.

The caregiver survey contained measures of caregiver role, HRQL and caregivers' perceptions of reciprocity and balance in the relationship, and relationship satisfaction. Demographic information was also collected.

#### Caregiver HRQL

The SF-36 was used to assess HRQL. This is a widely used measure in health research [32] that has been used with family caregivers of patients with stroke [33]. The SF-36 provides a general assessment of eight different health domains; physical functioning; role limitations due to physical problems; bodily pain; general health; vitality; social functioning; role limitations because of emotional problems; and mental health. Each is scored, summed and transformed to a scale ranging from 0 to 100, with 0 being the worst possible health state and 100 the best possible health state [31]. In addition, SF-36 Physical (PCS) and Mental (MCS) Component summary scales were computed according to standard scoring algorithms [34]. Internal consistency for the SF-36 PCS was α = .85, and for the SF-36 MCS α = .81.

#### Caregiver role

The Caregiver Reaction Assessment (CRA) was used to measure the perception of caregiving on four negative subscales (disrupted schedule, financial problems, lack of family support, and health problems), which we summed to form CRA negative dimension, and one CRA positive dimension (caregiver self-esteem subscale) [35]. Items are rated on five-point Likert scales ranging from "strongly disagree" to "strongly agree", with higher scores reflecting greater caregiver reaction. In general, the CRA has been found to be a reliable and valid measure [35]. In the present study the CRA negative subscales had reliabilities of α = .73 to α = .89. The internal consistency of the positive scale was α = .83. The CRA has been used to assess the caregiving role in caregivers of people with stroke [36,37].

#### Reciprocity

The quality and intensity of exchanges between the care recipient and caregiver were assessed by caregivers with the 22 item Caregiver Reciprocity Scale II (CRS II) [38]. The scale consists of four subscales representing different dimensions of reciprocity (warmth and regard, intrinsic rewards of giving, love and affection, and balance within family caregiving), where each item is rated on a 5-point scale ranging from 1 (strongly disagree) to 5 (strongly agree). Scores for the subscales are calculated by summing the items after reverse scoring selected items. Higher scores represent greater levels of perceived reciprocity. There is evidence to support the reliability and validity of the tool [38]. The internal consistency of three of the CRS subscales was acceptable ranging from α = .72 to α = .83. The reliability for the balance in family caregiving subscale, however, was low α = .66.

#### Perceived equity in the relationship

Caregivers' completed a single item question with a 5-point scale, based on the Hatfield Global Measure [39]. The item asks the respondent to describe the give and take in their relationship. The five response options range from "my partner is doing more for me than I am doing for him/her" (+ 2) to "my partner is doing a lot less for me than I am doing for him/her" (- 2). This question was developed by Kuijer et al. and has been found to be sensitive to perceived changes in equity, currently and before illness, among couples facing cancer [40]. Two items were used in the present study to assess caregivers' perceptions of balance in the relationship currently and prior to the stroke.

#### Relationship satisfaction

The Quality of Marriage Index (QMI) was used to evaluate caregivers' views on the degree of satisfaction in their relationship [41]. The measure is widely used and has excellent reliability (α = .97), discriminant and convergent validity [42]. This is a brief 6-item measure with seven response options ranging from "very strongly agree" to "very strongly disagree". The scale also includes one item that evaluates the degree of happiness in the relationship on a one to ten scale from "unhappy" to "perfectly happy". QMI scores tend to be positively skewed; therefore the scores were transformed following the procedure recommended by Norton [41]. In this study α = .94, indicating excellent reliability.

### Analysis

All data were checked to ensure that the test assumptions for the statistical analyses were met. Descriptive statistics were calculated to describe participants' HRQL, relationship satisfaction, reciprocity, balance in the relationship and caregiver role. To describe our sample in terms of their HRQL, we compared participants' SF-36 scores with data from a normative sample derived from US population norms [34]. Using Cohen's *d *to compare the standardized differences and interpreted the effect sizes as follows: .2 small, .5 medium, and .8 large.

To examine the relationship between the variables and address our hypotheses: (1) caregiver perceived reciprocity (CRS II) will be significantly related to CRA positive dimension and inversely related to CRA negative dimension, and (2) satisfaction with the relationship (QMI) will be related to the current balance in give and take, we used bivariate Pearson *r *correlations. To identify predictors of caregiver HRQL (SF-36) and CRA positive and negative dimensions, we conducted a series of multiple linear regression analyses entering variables simultaneously. Variables were selected based on the literature, clinical significance and *p*<0. 05. We anticipated that care recipient quality of life and negative aspects of the caregiver role would to be significant predictors of caregiver HRQL[1-8]. We did not enter caregiver HRQL as a predictor of caregiver role, as it was entered as a predictor of HRQL. Instead, the focus was the care recipient and the relationship dynamic. Relationship satisfaction and reciprocal exchanges have been identified as important in finding satisfaction in caregiving and for reducing the burdens associated with caregiving [10-12,18-22,27-29]. Based on the available literature therefore, we anticipated that quality of the relationship and reciprocity would predict positive aspects of the caregiver role, and care recipient quality of life and reciprocity, negative aspects of the caregiver role. Our a priori power analysis based on, at most, six predictors, using an alpha .05, power .80 and large effect size .35 (derived from the aforementioned literature) indicated that our sample size would need to be at least 46 participants [43].

To test our third hypothesis that caregivers who are underbenefiting in the current relationship will have scores on the CRA negative dimension than those who are equitable or overbenefiting from the relationship, we divided the group into two *underbenefiting *and *equitable/overerbenefiting *based on the balance in their current relationship. We then compared the two groups on CRA negative dimension using a t-test. Alpha was set at .05.

## Results

### Descriptive statistics

#### Caregiver health-related quality of life

Comparing descriptive statistics for caregiver HRQL to normative data from the general US population [34], we found that scores were lower for all SF-36 domains (Table 2). Notably, the largest differences were seen for limitations in physical role, followed by limitations in physical functioning and emotional role.

**Table 2:** Caregiver HRQL Scores on the SF-36 Compared With a Normative Sample

	**Study sample**		**Normative sample^1^**		
		
**SF-36 domains**	**Mean**	**SD**	**Mean**	**SD**	**Cohen's *d***
	
Limitations in physical functioning	70.52	24.71	84.15	23.28	.57
Limitations in physical role	50.96	43.15	80.96	34.00	.77
Bodily pain	71.24	27.10	75.15	23.69	.15
General health	64.64	23.45	71.95	20.34	.33
Vitality	56.18	19.34	60.86	20.96	.23
Limitations in social functioning	77.50	25.39	83.28	22.69	.24
Limitations in emotional role	62.65	42.82	81.26	33.04	.49
Mental health	70.55	18.24	74.74	18.05	.23

*Note*. ^1^Norms for the general US population (N = 2474)

#### Caregiver role

Caregivers' scores on the four negative CRA subscales were: disrupted schedule (*M *= 15.30, *SD = *5.03), financial problems (*M *= 7.0, *SD = *2.52), lack of family support (*M *= 11.81, *SD = *4.15) and caregiver health problems (*M *= 9.84, *SD = *3.50). The positive domain of caregiver self-esteem was *M *= 24.31 (*SD = *2.52) out of a possible 35.

#### Caregiver perceived reciprocity

Scores for each of the four subscales balance in family caregiving (*M *= 11.10, *SD = *2.70), intrinsic rewards of giving (*M *= 22.15, *SD = *2.67), warmth and regard (*M *= 25.40, *SD = *4.48), and love and affection (*M *= 17.55, *SD = *2.67), indicated that on average, perceived reciprocity was high for all of these domains.

#### Relationship satisfaction

In general the majority of participants were happy in their relationship with 50 (89.2%) rating between six (happy) and 10 (perfectly happy). Scores on the QMI indicated that in general participants were satisfied with their relationship.

#### Caregiver perceived equity in the relationship

When asked about the give and take in their current relationship, 34 (60.7%) reported that they were doing more for their partner then he/she was doing for them. Of these 23 (41.1%) indicated that they were doing a lot more for their partner. Only one participant indicated that there partner was doing slightly more for them. While 21 (37.5%) felt that the balance was equitably. This contrasts with the balance in the relationship prior to their partners' stroke where more than half felt the balance was equitable (57.1%) and 16 (28.6%) of the participants were overbenefiting. Only one participant was contributing a lot more and seven (12.5%) slightly more to the relationship than their partner was contributing.

### **Correlations between caregiver perceived reciprocity, caregiver role and relationship satisfaction**

None of the care recipient or caregiver demographics (age, gender, education, time since the stroke) were significantly associated with relationship satisfaction, perceived reciprocity or caregiver role. Table 3 contains a summary of the correlations between reciprocity, caregiver role and relationship satisfaction.

**Table 3:** Correlations between Caregiver Variables and Care Recipient Quality Of Life

	SF-36 (PCS)	SF-36 (MCS)	CRA (negative)	CRA (positive)
SF-36 (PCS)	1			
SF-36 (MCS)	.16			
CRA (negative)	-.50**	-.43**		
CRA (positive)	-.11	.26	-.24	
CRS II (warm)	-.14	.16	-.26	.16
CRS II (intrinsic)	-.13	-.19	-.02	.42**
CRS II (love)	-.12	-.12	.14	.31*
CRS II (balance)	.03	.08	-.41**	.29*
Current balance	-.02	-.04	-.29	.27
QMI	-.03	.28*	-.18	.44**
Care recipient SS-QOL	.45**	.47**	-.51**	.16

**Significant at the .01 level, *significant at the .05 level. SF-36 (PCS): Physical Component Summary score, SF-36 (MCS): Mental Component Summary score, CRA: Caregiver Reaction Assessment (positive and negative dimensions), CRS II: Caregiver Reciprocity Scale (four subscales: warmth and regard, intrinsic rewards of giving, love and affection, and balance within family caregiving), QMI: Quality of Marriage Index, Current balance: Single item rating of perceived equity in the relationship, SS-QOL: Stroke Specific Quality of Life Scale

Our hypothesis (1) based on equity theory that reciprocity would be related to positive and inversely related to negative aspects of the caregiver role were partially supported. CRA positive dimension scores were significantly correlated with three of the four CRS II scales (intrinsic rewards of giving, love and affection, and balance in family caregiving). However, there was little support for an inverse relationship between CRA negative dimension scores and reciprocity. The only association was an inverse relationship with balance in family caregiving (*r *= -.41, *p *< 0.01).

Hypothesis (2) which stated that caregiver satisfaction with the relationship would be related to the current balance of give and take in the relationship was not supported (*r *= 0.07, NS). Of note was the significant correlation (*r *= .59, *p *< 0.01) with previous balance in the relationship.

### Predictors of caregiver health-related quality of life

With the exception of an inverse relationship between SF-36 PCS and care recipient age (*r *= -.46 *p *< 0.01) and caregiver age (*r *= -.45 *p *< 0.01), none of the other demographics were significantly associated with caregivers' HRQL. As expected, care recipients' SS-QOL scores were significantly correlated with all the caregiver SF-36 subscales: physical functioning (*r *= .43, *p *< 0.01); role limitations due to physical problems (*r *= .47, *p *< 0.01); bodily pain (*r *= .49, *p *< 0.01); general health (*r *= .47, *p *< 0.01); vitality (*r *= .53, *p *< 0.01); social functioning (*r *= .55, *p *< 0.01); role limitations due to emotional problems (*r *= .39, *p *< 0.01); and mental health (*r *= .36, *p *< 0.01). There were also modest to strong inverse relationships between the CRA negative dimension and SF-36 PCS and MCS (Table 3).

We examined which factors were predictive of caregiver HRQL using the SF-36 PCS and MCS. Multiple linear regression analyses were conducted entering variables simultaneously into the model. Since care recipient and caregiver age were highly correlated (*r *= .97, *p *< 0.01), we only included care recipient age as this was more highly correlated with SF-36 PCS. The CRA negative domain, care recipient age and quality of life (SSQOL), were entered into the model to predict SF-36 PCS. SSQOL did not contribute significant independent variance (*t *= 1.53, NS) beyond that of care recipient age and CRA negative domain (Table 4). As we expected the CRA negative domain and SS-QOL to predict mental component summary scores, these variables were entered into the model. When entered together SS-QOL did not contribute significant variance (*t *= 1.25, NS) beyond that of CRA negative domain. As a single predictor is equivalent to the correlation, we did not produce models for SF-36 MCS (correlations in Table 3).

**Table 4:** Regression Models to Identify Predictors of Caregiver Role and Caregiver HRQL

	SF-36 (PCS)				CRA (positive)				CRA (negative)		
	***b***	***SE b***	**ß**		***b***	***SE b***	**ß**		***B***	***SE b***	**ß**

Constant	3.68	.72		Constant	13.61	2.85		Constant	5.14	.46	
Care recipient age	-.03	.01	-.40**	QMI	.12	.05	.33*	CRS II (balance)	-.10	.03	-.40**
CRA (negative)	-.77	.19	.-.43**	CRSII (intrinsic)	.31	.14	.29*	SSQOL	-.04	.01	-.44*
*F *(2, 48) 16.34**				*F *(2, 52) 9.46*				*F *(2, 50) 16.46**			
Adjusted *R*^2 ^.39				Adjusted *R*^2 ^.24				Adjusted *R*^2 ^.37			

**Significant at the .01 level, *significant at the .05 level. SF-36 (PCS): Physical Component Summary score, CRA (negative): Caregiver Reaction Assessment (negative domain score), SS-QOL: Stroke Specific Quality of Life Scale; CRS II Caregiver Reciprocity Scale subscales- CRS II (balance) balance within family caregiving, CRS II (intrinsic): intrinsic rewards of giving, QMI: Quality of Marriage Index.

### Predictors of caregiver role

To identify predictors of CRA positive and negative domains, we conducted multiple linear regression analyses. Relationship satisfaction (QMI) and three of the four CRS II scales (intrinsic rewards of giving, love and affection, and balance in family caregiving) that were significantly correlated at the bivariate level with the dependant variable were entered into the model to predict CRA positive domain. Only QMI and CRS II intrinsic rewards of giving significantly predicted independent variance accounting for 24% of the variance in the CRA positive domain (Table 4). For the CRA negative dimension two predictors were entered, the CRS II intrinsic, as this was the only reciprocity subscale significantly correlated with the dependant variable, and care recipient quality of life. These two predictors accounted for 31% of the variance in CRA negative dimension (Table 4).

### Inequity in the relationship and caregiver burden

Comparison on the CRA negative domain scores between caregivers who were currently *underbenefiting *(*M *= 2.72, SD = 0.55) and those who were *equitable/overbenefiting *(*M *= 2.38, SD = 0.71) indicated that there was a significant difference between the groups (*t *(54) = -2.03, *p *= 02). This finding supports hypothesis (3) that caregivers who are underbenefiting in the current relationship have higher levels of caregiver burden than those who are equitable or overbenefiting from the relationship.

## Discussion

This study set out to examine the relationships between HRQL, caregiver role, reciprocity, balance and relationship satisfaction in a sample of caregivers whose partner had experienced a stroke. Furthermore, using equity theory and reciprocity as a basis, we made several predictions regarding the relationships and likely outcomes.

Not surprisingly, following the family member's stroke there was a change in the balance of give and take in the relationship, with almost a third of caregivers' reporting that they were currently doing more for their partner than their partner was doing for them. This contrasts sharply with balance in the relationship prior to their partner's stroke, where the majority perceived the relationship as equitable or were actually overbenefiting from the relationship. From the perspective of equity theory balance is important in maintaining satisfaction in the relationship [25,44]. We found partial support for this as relationship satisfaction was associated with previous balance prior to the care recipients' stroke but not current balance in the relationship. This suggests that balance may be important generally in relationships but may become less important in terms of relationship satisfaction when one member is in need, for instance following illness such as a stroke. Indeed, perceptions of inequity may be less salient in high quality relationships as both members of the dyad are motivated to maintain a mutually supporting relationship [28].

However, with respect to the caregiver role, current balance was important, supporting our hypothesis that caregivers who are currently underbenefiting in the relationship have higher levels in the caregiver negative domain than those who are equitable or overbenefiting from the relationship. Similar findings have been reported in caregivers whose partners have cancer or multiple sclerosis [28].

In our study we found that there was a high degree of perceived reciprocity, with exchanges between members of the dyad rather than in one direction from caregiver to care recipient. Reciprocity was not only important in terms of relationship satisfaction but also in terms of the caregiver identifying positive aspects of their role. This finding supports previous studies where exchanges of an interpersonal nature have been found to still occur and be important for caregiver well-being [14,18,27,29]. One explanation for the associations is that strong relationships, characterized by mutuality and satisfaction may be derived from being able to help one another in times of need. Certainly, being able to maintain care recipients' dignity and self-esteem can be a source of satisfaction [45]. Another possible explanation is that those who are satisfied in their relationship are likely to be more invested in the relationship and this may enhance their perception of competence in the role as caregiver [28]. The findings add support to a smaller number of studies that have examined the caregiver-care recipient relationship following stroke [21,22]. They highlight the importance of health professionals including assessments of the context of care in terms of the relationship dynamics and how the caregiver is coping with this role. Also, the findings point to potential interventions aimed at encouraging reciprocal exchanges and helping caregivers find meaning in their role. Approaches to restoring the perception of equity, include encouraging reciprocal exchanges between care recipient and caregiver. Care recipients with stroke-related limitations that are limited in their ability to provide material tangible support, could be encouraged to offer reciprocal exchanges of a more emotional nature, such as affection and caring toward their caregiver. Indeed, there is some evidence to support counselling interventions directed at restoring the perception of equity and fostering reciprocity in couples facing cancer [46].

In our study, we found less support for our prediction that a lack of reciprocity would be inversely related to negative aspects of the caregiver role. Only balance in family caregiving was inversely related. A possible explanation for these contrasting findings, consistent with Gouldner's (1960) premise is that needs rather than reciprocity may prevail under certain conditions [47]. Under circumstances where the care recipient is ill and less able to reciprocate, caregivers may focus on care recipients' needs as they may come to dominate with little expectation of reciprocation. However, when there is reciprocation, these exchanges may positively affirm the caregivers' contributions and bolster self-esteem.

Caregivers' perceptions of caregiving in our study were comparable to other studies with caregivers of stroke survivors [36,37]. Like Teel, comparing the CRA scores with those of caregivers of a family member with cancer [48], indicates that caregiving for people with stroke is perceived as more negative, with higher scores on the negative subscales and lower scores on the positive subscale. Our findings also add to the literature in demonstrating that caregiving can be a positive experience for some [4,5,9-12] and that both rewards and burdens can co-exist [13]. Research directed toward an understanding of relationship factors that influence positive and negative role perceptions and caregiver outcomes are particularly important for designing interventions to promote the well-being of both members of the dyad. Possible avenues for further investigation include attachment between caregiver and care recipient and how these bonds influence motivations for responding to the needs of another during times of illness [9].

Comparing caregivers' HRQL to population norms, our sample had lower levels in all domains. These findings parallels others in showing that caregiving can negatively impact on quality of life [1-3]. The multiple regression analysis indicated that older care recipients and caregivers who are themselves older, who are caring for a partner with more impaired functioning and who perceive the caregiver role as having a greater negative impact have lower HRQL physical component summary scores. Similarly, caring for a partner with more impaired functioning and finding caregiving burdensome was associated with the HRQL mental component summary score. Therefore, health professionals caring for individuals following a stroke should be mindful of those caregivers likely to be most affected and implement strategies to support them in their role.

The study has several limitations. First the low response rate meant that the sample size was small, consisting of mainly female caregivers recruited through one stroke rehabilitation clinic setting. Therefore, the generalisability to male caregivers, and those from other settings cannot be determined. Furthermore, the extent to which the sample is biased cannot be determined, as no information was collected on non-responders. A larger more diverse sample would permit more sophisticated multivariate analyses including an analysis of gender and cultural variations. Second, our inclusion criteria meant that caregivers were providing care to stroke survivors whose cognitive ability was not significantly impaired. The experiences of caregivers of individuals with more significant stroke-related cognitive and communication impairment may be very different from the present sample. Third, the cross-sectional correlational design meant that we could not determine the directionality or determine causality between the variables. Future research would benefit from examining the caregiving relationship over time as caregiver-care recipients' transition to the changes brought about by the stroke.

## Conclusions

Our findings suggest that the context of caregiving is important in terms of the relationship, caregiver-care recipient exchanges, and health and well-being of caregivers. Therefore, a more comprehensive understanding of the caregiving situation should incorporate these aspects. This will place health professionals and researchers in a better position to develop and implement interventions aimed at improving the well-being of both members of the dyad as they adapt to the changes brought about by the stroke.

## List of abbreviations

CRA: Caregiver Reaction Assessment; CRS II: Caregiver Reciprocity Scale; FIM: Functional Independence Measure; Health-related quality of life; QMI: Quality of Marriage Index; SF-36 MCS-Mental Component Summary score; SF-36 (PCS)-Physical Component Summary score; SS-QOL: Stroke Specific Quality of Life Scale.

## Competing interests

The authors declare that they have no competing interests.

## Authors' contributions

CJM was responsible for the development, design, statistical analysis, interpretation of data and drafting of the manuscript. KGW contributed to the data collection, statistical analysis, and revision of the manuscript. LC contributed to the data collection, statistical analysis, interpretation of data and revision of the manuscript. CL contributed to the data collection, interpretation of data and revision of the manuscript. All authors read and approved the final manuscript.
